# Mapping the Bioenergetic Landscape of Oral Cancer Stem Cells: A Systematic Review

**DOI:** 10.1111/odi.70449

**Published:** 2026-08-03

**Authors:** Rodrigo Elísio de Sá, Rebeca Barbosa da Rocha, Benedito dos Santos Alves, Maria Eduarda de Araujo de Carvalho, Jayane da Silva Sousa, José Delano Barreto Marinho‐Filho, Ana Jérsia Araújo

**Affiliations:** ^1^ Laboratório de Cultura de Células Do Delta (LCCDelta) Universidade Federal Delta do Parnaíba, UFDPar Parnaíba PI Brazil

**Keywords:** cancer stem cells, mitochondria metabolism, mitochondrial role, oral cancer, therapeutic resistance

## Abstract

**Objectives:**

Oral squamous cell carcinoma (OSSC) remains highly lethal, with resistance largely driven by cancer stem cells (CSCs). Mitochondrial reprogramming supports CSC redox control, self‐renewal, and survival. This systematic review aimed to synthesize mitochondrial mechanisms sustaining CSCs in OSCC and evaluate their therapeutic relevance.

**Methods:**

Following PRISMA guidelines and a PROSPERO protocol (CRD420251088208), we searched major databases for studies investigating mitochondrial biogenesis, dynamics, mitophagy, or redox regulation in oral CSCs. Extracted data included molecular pathways, functional roles, and pharmacological targeting.

**Results:**

Ten studies were eligible. Evidence shows that mitochondrial remodeling, through PGC‐1α/ERRα‐mediated biogenesis, DRP1‐dependent fission, BNIP3/PINK1‐regulated mitophagy, and NRF2‐driven antioxidant signaling, supports metabolic flexibility and drug tolerance. These pathways activate stemness regulators, including EGFR/JNK/SOX2, YAP/CCN1, and SIRT1/PGC‐1α, promoting adaptation and recurrence. Pharmacological interventions targeting these nodes, such as DRP1 inhibitors, SIRT1 or ERRα antagonists, and mitophagy‐modulating agents (melatonin, verteporfin), showed potential to sensitize CSCs to conventional therapies.

**Conclusion:**

Mitochondria act as central regulators of CSC biology in OSCC and represent actionable therapeutic targets. Integrating mitochondrial dynamics, metabolism, and redox control provides a framework for overcoming resistance. Further preclinical and clinical studies are needed to translate these vulnerabilities into effective OSCC therapies.

**Trial Registration:**

PROSPERO, CRD420251088208

## 1 Introduction

Oral squamous cell carcinoma (OSCC) is an aggressive malignancy arising from the buccal squamous epithelium (Solomon et al. 2018). It is the seventh most common cancer worldwide, representing about 2% of all cases and deaths (Olmedo et al. 2023). Treatment varies by stage and patient profile: early OSCC is mainly surgical, whereas advanced disease requires combined surgery, radiotherapy, and chemotherapy (Bossi et al. 2014; Mohamad et al. 2023). Most patients present with stage III–IV disease, compromising quality of life (Chamoli et al. 2021). Late diagnosis contributes to poor outcomes, with a five‐year survival of ~50%, underscoring its persistent clinical burden (Ahmad et al. 2021; Weckx et al. 2020). Despite therapeutic advances, treatment resistance remains a major obstacle to improving prognosis (Sá et al. 2026).

A key advance in cancer biology was recognizing that tumors are heterogeneous networks, with cancer stem cells at the hierarchy apex (Jagust et al. 2019). CSCs can self‐renew and differentiate, driving tumor initiation, progression, and therapy resistance (Phi et al. 2018). They also exhibit survival mechanisms that enable treatment evasion and tumor recurrence (Shibata and Hoque 2019).

Among the principal CSC markers in OSCC, CD44 and aldehyde dehydrogenase (ALDH) are associated with stemness, metabolic adaptation, and therapeutic resistance (Praharaj et al. 2023). CD44 interacts with the xCT transporter to promote glutathione synthesis and NRF2 activation, contributing to redox balance, preservation of mitochondrial membrane potential, ROS control, and mitochondrial protection under hypoxic and chemotherapy‐induced stress conditions, thereby supporting CSC survival and metabolic plasticity (Saluja et al. 2020).

Similarly, ALDH activity is associated with mitochondrial bioenergetics and detoxification of reactive aldehydes generated during oxidative metabolism. Elevated ALDH expression contributes to the removal of toxic metabolic byproducts, maintenance of redox homeostasis, and adaptation to hypoxic tumor niches, thereby supporting CSC survival under metabolic stress and promoting resistance to chemotherapy and radiotherapy (Dhumal et al. 2022). In parallel, alterations in mitochondrial dynamics, particularly DRP1‐mediated mitochondrial fission and adaptive mitophagy, have emerged as promising therapeutic targets, as inhibition of these pathways may impair mitochondrial quality control, reduce stemness maintenance, and sensitize CSCs to conventional therapies (Zhang et al. 2023).

Mitochondria regulate metabolism, cell death, and stress responses in cancer stem cells, supporting stemness and therapeutic resistance (Garimella et al. 2023; Zhang et al. 2023). In OSCC, mitochondrial remodeling promotes metabolic plasticity and CSC survival in hypoxic niches, while altered dynamics regulate signaling and mitophagy (Tan et al. 2024, 2025). Mitochondrial activity is also associated with redox balance and maintenance of stem‐like phenotypes (Vasan et al. 2020; Wu, Chen, et al. 2024; Wu, Xiao, et al. 2024). Dysregulation of mitochondrial fission, mitophagy, and antioxidant signaling contributes to proliferation, invasiveness, and therapy resistance in OSCC (Lin et al. 2021). Additionally, mitochondrial metabolites may act as epigenetic cofactors reinforcing stemness and aggressive phenotypes, highlighting mitochondria as potential therapeutic targets (Du et al. 2025).

Despite existing evidence, this review aims to synthesize current knowledge on the role of mitochondria in cancer stem cells in OSCC. It examines key molecular mechanisms, their implications for tumor progression and therapeutic resistance, and identifies gaps that may guide future research and therapeutic strategies.

## 2 Methods

### 2.1 Reporting Standards

This systematic review was prepared in accordance with the *Preferred Reporting Items for Systematic Review and Meta‐Analysis Protocols* (PRISMA) guidelines (Page et al. 2021). The review protocol was registered in the International Prospective Register of Systematic Reviews (PROSPERO) [registration number: CRD420251088208].

### 2.2 Search Strategies and Article Selection

Scientific articles on mitochondria in cancer stem cells in oral squamous cell carcinoma were retrieved from Web of Science, PubMed, Embase, and Scopus using keywords such as “oral squamous cell carcinoma,” “cancer stem cell,” “mitochondria,” “mitochondrial metabolism,” “mitochondrial dynamics,” “therapeutic target,” and “oxidative phosphorylation” (Table S1). Terms were used alone or combined with AND/OR for a comprehensive search, last performed in September 2025. Duplicates were removed, and study selection occurred in two stages, title/abstract screening and full‐text review, by three blinded reviewers, with discrepancies resolved by a fourth. Reference lists of included studies were also screened for additional articles.

### 2.3 Inclusion and Exclusion Criteria

Inclusion criteria covered in vitro and in vivo studies investigating cancer stem cells in oral squamous cell carcinoma. Only original research with primary data was included. Eligible studies assessed mitochondrial aspects, including metabolism, biogenesis, dynamics, signaling, apoptosis, and reactive oxygen species. Human‐ or animal‐derived models, cell lines, organoids, murine models, or clinical samples were considered if CSC isolation or enrichment methods were used, such as spheroid formation assays or the evaluation of CSC markers, including CD44, CD133, and ALDH activity, as well as stemness‐associated transcription factors (e.g., SOX2, OCT4, NANOG, and BMI‐1). No restrictions on language or publication year were applied. Broad mitochondrial‐ and CSC‐related descriptors were used in the search strategy to maximize sensitivity, while markers such as CD44, CD133, ALDH, SOX2, OCT4, NANOG, and BMI‐1 were applied as phenotypic validation criteria during study selection.

Excluded studies included reviews, editorials, letters, conference abstracts, theses, and dissertations. Also excluded were studies on mitochondria in oral squamous cell carcinoma without focus on CSCs, CSCs from other cancers without OSCC, or CSCs in oral cancer not assessing mitochondrial aspects. Studies with insufficient or speculative data were also excluded.

### 2.4 Assessment of Risk of Bias

For the included articles, methodological quality was assessed using the Quality Assessment Tool for in vitro Studies (QUIN) and the SYRCLE risk‐of‐bias tool for in vivo studies. The QUIN tool evaluates 12 parameters: (1) clearly defined goals/objectives, (2) sample size calculation, (3) sampling method, (4) comparison group details, (5) methodological description, (6) operator details, (7) randomization, (8) outcome measurement, (9) outcome assessor details, (10) blinding, (11) statistical analysis, and (12) presentation of results. Each parameter was scored according to study specifications (adequately specified = 2; inadequately specified = 1; not specified = 0) (Sheth et al. 2024). The final score was calculated according to the following formula:

$$\text{Final Score}=\left(\text{Total}\times 100\right)/\left(2\times \;\text{number of applicable criteria}\right).$$

The SYRCLE risk‐of‐bias tool comprises 10 items assessing methodological steps in animal studies: (1) sequence generation, (2) baseline characteristics, (3) allocation concealment, (4) random housing, (5) blinded animal interventions, (6) random outcome assessment, (7) blinding of outcome assessors, (8) incomplete outcome data, (9) selective outcome reporting, and (10) other bias sources. A “yes” indicates low risk of bias, “no” indicates high risk, and “unclear” denotes insufficient information to determine risk (Askari et al. 2024; Hooijmans et al. 2014).

### 2.5 Data Collection Process

A pilot spreadsheet was completed with data from included articles and independently analyzed by three reviewers, with discrepancies resolved by a fourth. The data collection spreadsheet captured the following information: article title, year, study type (in vitro, in vivo), experimental model, cell type, CSC markers, mitochondrial functions assessed, signaling pathways, roles in tumor progression and therapeutic resistance, proposed therapeutic targets, tested interventions, main results, and study limitations.

### 2.6 Synthesis Method

The descriptive analysis was conducted considering the heterogeneity of results reported across the included studies. Accordingly, the data were grouped based on the type of analysis performed.

## 3 Results

### 3.1 Study Selection

Initially, a total of 798 articles were identified in our systematic search; after an initial screening and removal of duplicates, 415 articles were analyzed. Following full‐text assessment, ten studies were included in the review. An overview of the study selection process is presented in Figure 1.

**FIGURE 1:**
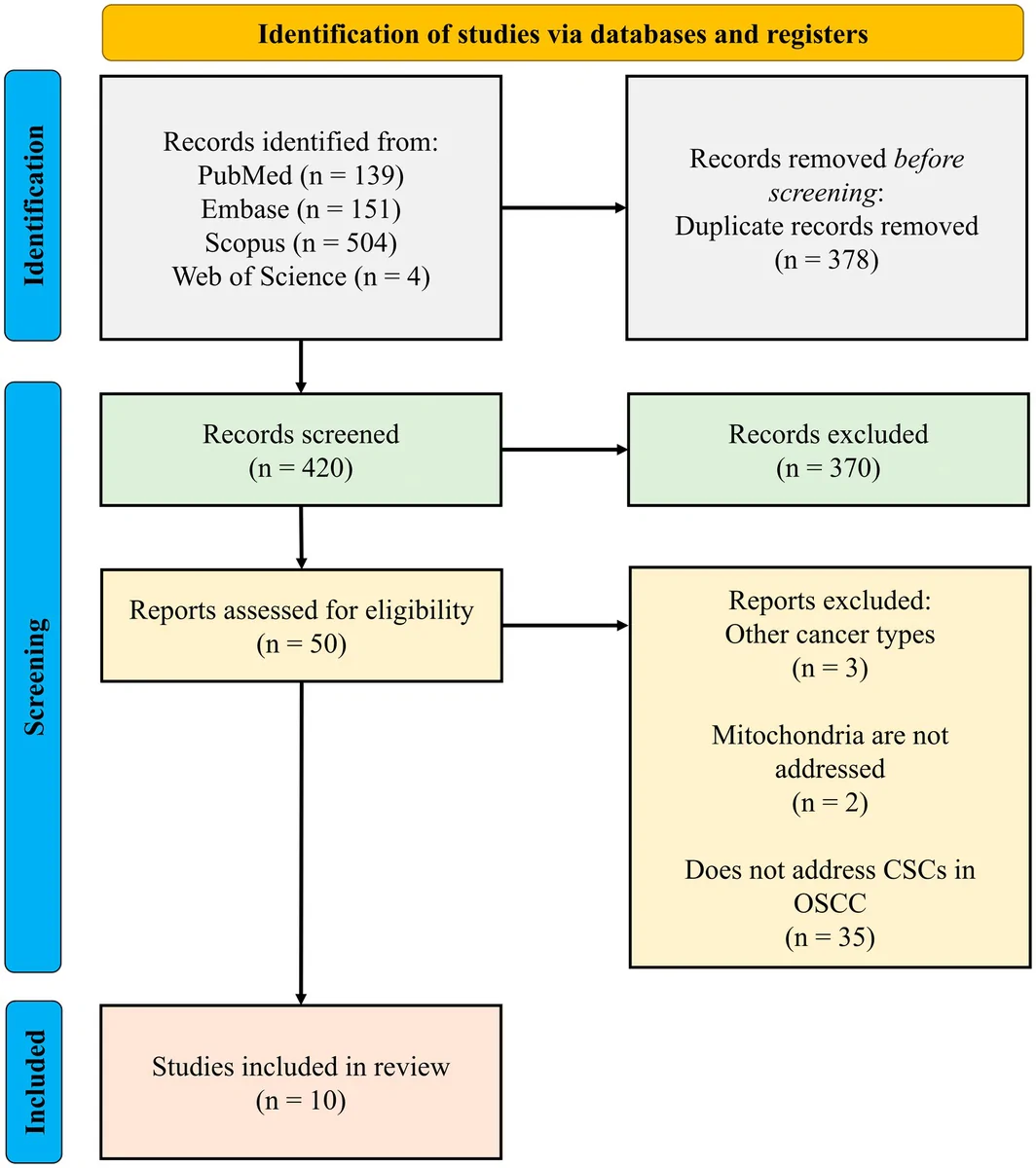
Flowchart of the study selection process according to the Preferred Reporting Items for Systematic Reviews and Meta‐Analyses (PRISMA) guidelines.

Among the experimental studies included in the review, 9 adopted in vitro experimental assays involving cell cultures, and 4 employed in vivo preclinical assays with mice. Three studies used both methodologies. Table 1 summarizes the main characteristics and results of the studies.

**TABLE 1:** The baseline characteristics and the main results of included studies.

Author/year	Study model	CSC markers	Outcome measures	Main results
Wu, Chen, et al. 2024	In vitro: TSCC cell lines (CAL27, SAS), 3D spheroids	Not specified (CSC‐enriched spheroids)	Lipid metabolism (UPLC‐MS), mitochondrial morphology, lipid droplets	Mitochondrial dynamics modulate lipid metabolism; inhibition of fission reduced lipid droplets and altered lipid species, affecting CSC metabolism.
Li et al. 2025	In vitro: OSCC cell line OECM‐1 and derived CSCs under hypoxia	Sphere‐derived CSCs (EMT/stemness proteins)	Sphere formation, migration/invasion, EMT markers, mitochondrial metabolism, mitophagy (BNIP3)	Hypoxia activated BNIP3‐driven mitophagy, ↑ OXPHOS and stemness, promoting CSC survival and invasiveness; BNIP3 silencing reduced CSC features.
Shimonosono et al. 2021	In vitro and in vivo: 3D organoids (HNSCC, ESCC), PDX, xenografts	CD44^high	Organoid formation, apoptosis, autophagy markers, tumor growth	Ethanol metabolism induced oxidative stress/apoptosis in non‐CSCs; CD44^high CSCs survived
Wang et al. 2024	In vitro and in vivo: OSCC cell lines (HSC3, HN12), patient‐derived organoids/xenografts	ALDH^high, SOX2, NANOG, OCT4	Mitochondrial morphology, α‐KG metabolism, histone methylation, ferroptosis sensitivity	DRP1 inhibition → mitochondrial elongation, ↓ stemness, ↑ glutaminolysis, α‐KG‐mediated epigenetic changes, ↑ ferroptosis susceptibility.
Shin et al. 2022	In vivo: HNSCC cell lines (SCC‐25, SCC‐1, SCC‐104), spheres, ROS‐low CSCs	CD24, CD44, CD133	Viability, apoptosis, sphere formation, ROS, mitophagy markers (PINK1, Parkin), migration	Melatonin + verteporfin synergistically induced apoptosis, ↓ stemness, ↓ mitophagy, impaired mitochondrial function, ↓ migration.
Saluja et al. 2020	Ex vivo/In vitro primary: Primary OSCC cultures (15 patients), spheroids	CD44, CD133, CD147, CD166, SOX2	Quiescence (Ki67), ROS, glutathione, apoptosis, drug synergy	Dyrk1b inhibition ↓ quiescence, sensitized CSCs to apoptosis; combo with Topo‐II and HDAC inhibitors synergistically induced mitochondrial stress and eliminated CSCs.
Praharaj et al. 2023	In vitro: OSCC cell lines (FaDu, CAL‐33), CD44+ sorted CSCs, spheres	CD44, SOX2, NANOG, OCT4	Autophagy (ATG5, BECN1), NRF2 signaling, ROS, cisplatin sensitivity	Cisplatin ↑ CSCs via autophagy; dual inhibition of autophagy + NRF2 ↑ mtROS, ↓ stemness, sensitized CSCs to cisplatin‐induced apoptosis.
Yuan et al. 2023	In vitro and in vivo: OSCC cell lines (HSC‐3, Ca9‐22, OECM‐1), xenograft in mice, patient tissues	SOX2	Cell growth, tumor sphere/colony formation, migration/invasion, OCR (Seahorse), survival (Kaplan–Meier)	IL‐1RA overexpression enhanced stemness, tumor growth, migration, and radioresistance via mitochondrial metabolism → EGFR/JNK/SOX2 axis.
Miao et al. 2024	In vitro and vivo: OSCC cell lines (CAL27, HSC3), MCTS, single‐cell RNA‐seq (patients)	SOX2, NANOG, OCT4	Metabolomics, transcriptomics, scRNA‐seq	CSCs showed ↓ metabolic activity vs. differentiated cells (↓ glycolysis, glutathione, lipid metabolism), suggesting resistance to metabolic‐targeted therapy.
Heawchaiyaphum et al. 2023	In vitro and in vivo: EBV‐positive OSCC cells (SCC25, HSC1), xenografts	CD44, CD133	mtDNA copy number, glycolysis (GLUT1, LDHA, PDK1), lactate production, tumor growth	EBV induced Warburg effect, ↑ glycolysis and lactate, ↑ CD44/CD133 expression, enhancing tumorigenicity and CSC features.

### 3.2 Models for CSC Isolation and Enrichment

Collectively, the included studies predominantly relied on in vitro experimental models to investigate mitochondrial regulation in OSCC CSCs, while several incorporated complementary in vivo validation through xenografts, spheroids, organoids, or functional animal assays. Evidence derived from these preclinical systems consistently supports the relevance of mitochondrial remodeling in CSC maintenance, therapeutic resistance, and metabolic adaptation. However, no studies employed immunocompetent models or directly performed functional CSC isolation from native clinical tumor tissues, limiting validation within physiologically complex tumor microenvironments.

Most studies combined conventional OSCC cell lines, including HSC‐3, CAL27, SAS, SCC‐25, and FaDu, with advanced experimental approaches such as three‐dimensional spheroids, organoid cultures, lipidomics, and bioinformatics analyses. Patient‐derived primary cells and clinically relevant samples were also incorporated in some investigations to strengthen translational relevance. Overall, these approaches consistently demonstrated that CSC‐enriched populations exhibit enhanced stemness, redox regulation, metabolic plasticity, and resistance‐associated phenotypes across distinct experimental settings.

CSC enrichment and phenotypic validation were commonly achieved through spheroid formation assays and characterization of ALDH^high/CD44^+ subpopulations, frequently combined with stemness‐associated markers such as CD133, SOX2, OCT4, NANOG, and BMI‐1. Across studies, these models were consistently associated with increased tumorigenic potential, metabolic adaptation, and resistance to oxidative and therapeutic stress. In vitro findings were further supported by xenograft and organoid models, particularly regarding the relationship between mitochondrial plasticity and aggressive CSC phenotypes.

Several studies also incorporated microenvironment‐related stressors, including hypoxia, ROS modulation, inflammatory signaling, and EBV infection, to investigate how environmental conditions influence mitochondrial behavior and CSC maintenance. These findings suggest that metabolic plasticity and mitochondrial adaptation are not restricted to isolated cell cultures but are also linked to stress‐related conditions that partially mimic the tumor niche. Nevertheless, evidence regarding stromal interactions and immune‐dependent regulation remains limited due to the predominance of simplified preclinical models.

### 3.3 Mitochondrial Remodeling and Signaling: Drivers of Tumor Progression and Therapeutic Resistance

The included studies demonstrate that OSCC CSCs undergo extensive mitochondrial remodeling to sustain metabolic plasticity, stemness maintenance, and therapeutic resistance. Across distinct experimental models, including CSC‐enriched cultures, spheroids, organoids, and xenograft assays, mitochondrial alterations consistently involved changes in biogenesis, dynamics, mitophagy, redox regulation, and signaling integration. These findings indicate that mitochondria function not only as energy suppliers but also as central regulators of CSC adaptation and aggressiveness.

Most mechanistic evidence was derived from in vitro models exposed to conditions mimicking hypoxia, oxidative stress, and nutrient deprivation, whereas in vivo validation was mainly limited to xenograft‐based approaches. Despite this limitation, the studies consistently support the concept that mitochondrial plasticity enables CSC survival within hostile tumor niches and contributes to therapy resistance.

In both clinical samples and experimental models, CD44^high CSC populations exhibited increased mitochondrial mass, membrane potential, and ROS production maintained by the CD44‐xCT‐NRF2 axis, supporting antioxidant defense and adaptation to oxidative stress. Similarly, enhanced mitochondrial biogenesis mediated by the PGC‐1α/ERRα axis promoted oxidative phosphorylation and favored CSC persistence under hypoxic and nutrient‐poor conditions (Figure 2A). Together, these findings suggest that mitochondrial biogenesis and redox regulation are critical for maintaining CSC fitness under microenvironmental stress.

**FIGURE 2:**
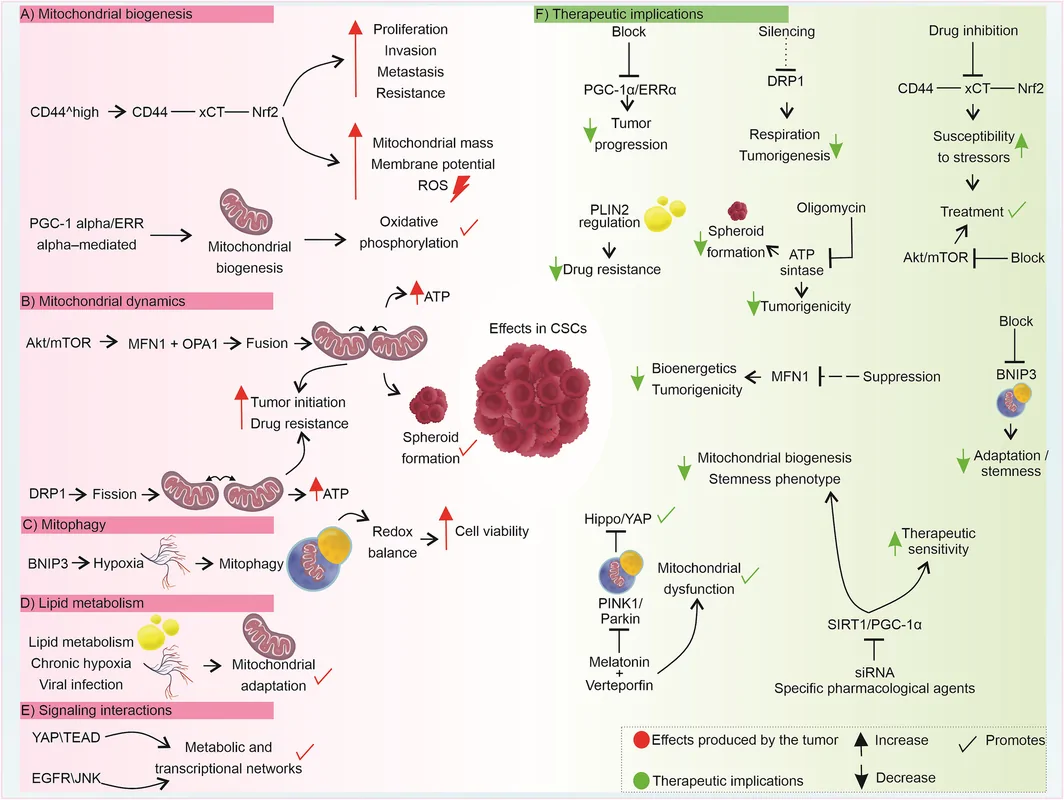
Schematic representation of mitochondrial mechanisms driving CSC maintenance, metabolic plasticity, redox regulation, and therapeutic resistance in OSCC. (A) *Mitochondrial biogenesis*: Activation of the PGC‐1α/ERRα axis and CD44–xCT–Nrf2 signaling enhances mitochondrial biogenesis, oxidative phosphorylation (OXPHOS), and redox balance, leading to increased mitochondrial mass, ATP production, and ROS levels, thereby promoting proliferation, invasion, metastasis, and therapeutic resistance. (B) *Mitochondrial dynamics*: Fusion (MFN1 and OPA1) and fission (DRP1) regulate mitochondrial morphology and bioenergetics, supporting ATP production, spheroid formation, tumor initiation, and drug resistance in CSCs. (C) *Mitophagy*: Under hypoxic conditions, BNIP3‐mediated mitophagy contributes to mitochondrial quality control and redox homeostasis, supporting CSC adaptation to stress. (D) *Lipid metabolism and microenvironmental inputs*: Lipid metabolism, chronic hypoxia, and viral infection promote mitochondrial adaptation and metabolic plasticity, reinforcing CSC survival and function. (E) *Signaling interactions*: Oncogenic pathways, including YAP/TEAD and EGFR/JNK, modulate metabolic and transcriptional networks that sustain CSC phenotypes. (F) *Therapeutic implications*: Targeting mitochondrial pathways disrupts CSC functional properties through multiple strategies, including inhibition of PGC‐1α/ERRα (reducing tumor progression), silencing of DRP1 (impairing respiration and tumorigenesis), blockade of CD44–xCT–Nrf2 signaling (increasing susceptibility to stressors), modulation of PLIN2 (reducing drug resistance), inhibition of BNIP3‐mediated mitophagy (compromising CSC adaptation and stemness), and inhibition of ATP synthase by oligomycin (reducing ATP production and tumorigenicity). Additional approaches include targeting Akt/mTOR and SIRT1/PGC‐1α signaling, enhancing therapeutic sensitivity. Arrows indicate activation or increase, whereas blunt‐ended lines indicate inhibition or blockade. Red symbols represent tumor‐promoting effects, while green symbols indicate therapeutic inhibition or reduction.

Mitochondrial dynamics also emerged as a central mechanism regulating CSC behavior. Increased fusion mediated by MFN1 and OPA1, frequently associated with Akt/mTOR signaling, enhanced ATP production, stemness maintenance, and tumorigenic capacity, whereas inhibition of fusion reduced bioenergetic efficiency and aggressiveness. Conversely, DRP1‐mediated fission contributed to metabolic adaptation and resistance, indicating that the balance between fusion and fission governs mitochondrial flexibility and CSC survival (Figure 2B). Importantly, evidence from both spheroid assays and xenograft models supports the association between altered mitochondrial dynamics and tumor progression.

Adaptation to hostile microenvironments was strongly linked to mitophagy and metabolic reprogramming. Under hypoxic conditions, BNIP3‐mediated mitophagy preserved redox homeostasis and prevented the accumulation of dysfunctional mitochondria, thereby sustaining CSC viability and resistance to stress (Figure 2C). In parallel, CSCs displayed marked metabolic heterogeneity, with subsets alternating between oxidative phosphorylation and glycolytic phenotypes according to environmental conditions. Although this metabolic plasticity was primarily demonstrated in vitro, the findings suggest that similar adaptive mechanisms may operate within hypoxic tumor niches.

Integrated regulation between lipid metabolism and mitochondrial function was also consistently observed. PLIN2‐mediated lipid droplet accumulation appeared to support mitochondrial redox balance and provide metabolic substrates for CSC adaptation, reinforcing resistance to stress and therapeutic injury (Figure 2D). In addition, extrinsic factors such as chronic hypoxia and viral infection promoted mitochondrial reprogramming and CSC‐like phenotypes, highlighting the influence of the tumor microenvironment on mitochondrial behavior and bioenergetic adaptation.

The studies further indicate that mitochondria operate as signaling hubs connecting metabolism to transcriptional regulation. Activation of YAP/TEAD signaling enhanced mitochondrial quality control and CSC plasticity, whereas EGFR/JNK signaling linked external growth stimuli to the expression of pluripotency‐associated factors such as SOX2 (Figure 2E). These interactions position mitochondria at the center of the metabolic and transcriptional networks sustaining CSC identity, tumor progression, and resistance.

Taken together, the available evidence indicates that mitochondrial remodeling in OSCC CSCs functions as an interconnected regulatory network rather than isolated pathways. Coordinated alterations in biogenesis, mitochondrial dynamics, mitophagy, lipid metabolism, and signaling collectively sustain bioenergetic flexibility, adaptation to microenvironmental stress, tumor aggressiveness, and therapeutic resistance. Although most evidence remains limited to preclinical models, the consistency of findings across different experimental systems supports mitochondria as central regulators of OSCC CSC biology and promising therapeutic targets.

### 3.4 Therapeutic Implications and Intervention Strategies

Collectively, the included studies indicate that mitochondrial metabolism represents a central therapeutic vulnerability in OSCC CSCs. Evidence derived predominantly from in vitro models, complemented by organoid and xenograft validation, consistently demonstrates that disruption of mitochondrial plasticity compromises stemness maintenance, tumorigenic potential, and therapy resistance (Figure 2F).

Several studies converged on the importance of redox regulation and mitochondrial biogenesis as critical adaptive mechanisms in CSC survival. Inhibition of the CD44‐xCT‐NRF2 axis reduced antioxidant defenses and increased sensitivity to oxidative stress, suggesting that targeting redox homeostasis may enhance the efficacy of conventional therapies (Saluja et al. 2020). Similarly, suppression of the PGC‐1α/ERRα and SIRT1/PGC‐1α pathways impaired oxidative phosphorylation, reduced stem‐like phenotypes, and increased therapeutic sensitivity, reinforcing the dependence of CSCs on mitochondrial biogenesis and metabolic adaptation (Li et al. 2025; Wu, Chen, et al. 2024; Wu, Xiao, et al. 2024).

Mitochondrial dynamics also emerged as consistent therapeutic targets across multiple experimental models. Studies evaluating MFN1 and DRP1 demonstrated that modulation of fusion and fission directly affects mitochondrial respiration, tumorigenicity, and resistance phenotypes. In particular, DRP1 silencing and MFN1 inhibition reduced bioenergetic flexibility and sensitized CSCs to therapeutic stress, highlighting mitochondrial dynamics as actionable regulators of CSC maintenance (Li et al. 2025; Wang et al. 2024).

Adaptive mitophagy was similarly associated with CSC survival under hypoxic and metabolically adverse conditions. In vitro and xenograft studies showed that inhibition of BNIP3‐ and PINK1/Parkin‐mediated mitophagy impaired migration, invasion, stemness maintenance, and cisplatin resistance, primarily by disrupting mitochondrial quality control rather than inducing direct cytotoxicity (Li et al. 2025; Shin et al. 2022).

Pharmacological interventions further supported the therapeutic relevance of mitochondrial targeting. Melatonin combined with verteporfin induced mitochondrial dysfunction and inhibited Hippo/YAP signaling, whereas oligomycin reduced viability, spheroid formation, and tumorigenicity by blocking oxidative phosphorylation (Shin et al. 2022; Wu, Chen, et al. 2024; Wu, Xiao, et al. 2024). In parallel, studies involving PLIN2 suggested that lipid metabolism contributes to mitochondrial adaptation and resistance to oxidative stress, indicating that lipid‐mitochondria interactions may represent additional therapeutic opportunities (Praharaj et al. 2023).

Importantly, multi‐omics analyses revealed marked metabolic heterogeneity among CSC populations, with subsets exhibiting either glycolytic or OXPHOS‐dependent phenotypes (Miao et al. 2024). These findings indicate that metabolic dependencies may vary according to microenvironmental conditions and cellular states, reinforcing the need for combinatorial therapeutic strategies capable of targeting distinct metabolic subpopulations simultaneously.

Overall, current evidence positions mitochondrial biogenesis, dynamics, mitophagy, redox regulation, and metabolic plasticity as interconnected therapeutic targets in OSCC CSCs. However, most available evidence remains restricted to preclinical models, with limited validation in native tumor microenvironments and clinically relevant systems (Table 2).

**TABLE 2:** Integrative summary of mitochondrial pathways and their functional roles in CSCs of OSCC.

Mitochondrial pathway/regulator	Mitochondrial function	Functional impact on CSCs	Therapeutic implication	Key studies
CD44‐xCT‐Nrf2 axis	Redox homeostasis	Maintains antioxidant capacity and protects against cellular stress	Targeting oxidative defenses may sensitize CSCs to therapy	Saluja et al. 2020
PGC‐1α/ERRα	Transcriptional regulation of mitochondrial biogenesis and OXPHOS	Promotes stemness and metabolic fitness	Inhibition reduces stem‐like traits and enhances chemosensitivity	Li et al. 2025; Wu, Chen, et al. 2024; Wu, Xiao, et al. 2024
SIRT1/PGC‐1α	Upstream regulation of mitochondrial biogenesis (via deacetylation)	Sustains stemness phenotype and metabolic activity	Inhibition reduces CSC properties and increases drug sensitivity	Li et al. 2025; Wu, Chen, et al. 2024; Wu, Xiao, et al. 2024
MFN1 (fusion)	Mitochondrial dynamics (fusion)	Supports bioenergetics and tumorigenicity	Suppression reduces tumor growth potential	Li et al. 2025; Wang et al. 2024
DRP1 (fission)	Mitochondrial dynamics (fission)	Regulates respiration and CSC maintenance	Silencing reduces tumorigenesis and metabolic activity	Li et al. 2025; Wang et al. 2024
BNIP3‐mediated mitophagy	Mitophagy (mitochondrial quality control under hypoxia)	Supports CSC adaptation and stemness under hypoxic stress	Inhibition impairs CSC fitness and stemness, without consistent evidence of direct cytotoxicity	Li et al. 2025
PINK1/Parkin pathway	Regulation of mitophagy	Maintains mitochondrial integrity and CSC stemness	Blockade reduces stemness and may overcome drug resistance	Shin et al. 2022
ATP synthase (Oligomycin)	Oxidative phosphorylation (ATP production)	Supports CSC viability, spheroid formation, and tumorigenicity	Inhibition reduces viability and tumorigenic potential	Wu, Chen, et al. 2024; Wu, Xiao, et al. 2024
PLIN2	Lipid metabolism—mitochondria crosstalk	Supports oxidative stress adaptation	Targeting may reduce resistance and metabolic flexibility	Praharaj et al. 2023
Metabolic heterogeneity (multi‐omics)	Regulation of energy metabolism (OXPHOS vs. inactive states)	Defines distinct CSC metabolic subpopulations	Reveals potential for targeted metabolic strategies	Miao et al. 2024

### 3.5 Risk of Bias Assessment

Methodological risk of bias in the 10 included articles was assessed using the QUIN tool for in vitro studies and the SYRCLE tool for in vivo studies. Each article was examined and scored based on experimental reliability criteria. Among in vitro studies, none showed a high risk of bias. Only Miao et al. (2024) was classified as low risk, due to methodological clarity, replication, outcome standardization, and robust statistics. Common issues in other in vitro studies included missing sample size calculations, lack of assessor blinding, and insufficient randomization details, essential to reduce variability in outcomes (Table S2).

In vivo studies showed a predominance of moderate risk of bias, with none classified as low risk (Table S3). For selection bias, most reported sequence generation, but allocation concealment and baseline similarity were often missing. Performance bias was common due to lack of blinding of caregivers and operators, potentially affecting experimental procedures. Detection bias was also present, as no studies reported blinding for outcome assessment and few used random outcome evaluations. Attrition bias was generally low, with most studies reporting data for all animals. Selective reporting bias appeared low, as outcomes in the methods were usually presented in the results. Overall, lack of formal randomization, blinding, and sampling descriptions remains a common limitation in both in vitro and in vivo studies (Tables S3 and S4).

## 4 Discussion

This review highlights the role of cancer stem cells in oral squamous cell carcinoma, particularly their involvement in tumor progression, therapeutic resistance, and mitochondrial regulation. Mitochondrial dynamics, especially the fission–fusion balance, emerge as central mechanisms controlling CSC plasticity, survival, and pro‐tumorigenic signaling, thereby contributing to tumor aggressiveness.

In oral cancer cell models, activation of Dynamin‐Related Protein 1 (DRP1) is associated with proliferation, spheroid formation, and increased resistance to oxidative stress (Zhang et al. 2023). These findings suggest that mitochondrial fission serves as an adaptive mechanism, enabling CSCs to generate heterogeneous cell populations capable of responding to distinct conditions such as hypoxia or nutrient deprivation (Jin et al. 2022).

However, mitochondrial fusion appears to be related to more differentiated and less proliferative stages, indicating that the balance between fission and fusion may act as a metabolic regulator in the transition between quiescence, cellular self‐renewal, and specialization in CSCs (Di Gregorio et al. 2022). This process has already been described in other cancer models, such as glioblastoma and breast cancer, supporting the notion that CSCs from various tumors share common mitochondrial relationships (Huang et al. 2025).

In addition to fission and fusion, mitophagy emerges as an important mechanism for mitochondrial balance in CSCs (Huang et al. 2025). Activation of BNIP3/BNIP3L and PINK1/Parkin indicates selective removal of damaged mitochondria to sustain oxidative metabolism. This preserves mitochondrial function and supports CSC proliferation and stemness (Onishi et al. 2021).

The hypoxic tumor microenvironment is a common feature in cancer (Chen et al. 2023). Mitophagy is key for CSC survival, eliminating dysfunctional mitochondria and reducing ROS, thereby conferring a selective advantage under stress (Napolitano et al. 2021). Reduced mitochondrial ROS links mitochondrial biology to therapeutic resistance by limiting the efficacy of oxidative stress–based therapies (Kowalczyk et al. 2021). CSC metabolic plasticity, enabling shifts between glycolysis and OXPHOS, also represents a vulnerability, as dual inhibition disrupts compensation, depletes ATP, and induces mitochondrial dysfunction and cell death (Sheng et al. 2024).

Mitochondrial reprogramming in oral carcinoma is driven by hypoxia, oral microbiota dysbiosis, and tobacco‐ and alcohol‐derived metabolites, which increase oxidative stress and stabilize HIF‐1α, favoring aerobic glycolysis. Altered mitochondrial dynamics and redox homeostasis then sustain tumor bioenergetics and promote apoptosis resistance and aggressiveness (Sheng et al. 2024; Wang et al. 2022).

Another aspect highlighted by the included studies was the relationship between mitochondrial metabolism and nuclear gene regulation. OSCC CSCs showed clear metabolic plasticity, alternating between aerobic glycolysis and oxidative phosphorylation (OXPHOS) according to nutrient and oxygen availability. This “metabolic switching” supports survival and activates signaling pathways essential for the stemness phenotype, such as EGFR/JNK/SOX2, YAP/CCN1, and NRF2 (DeBlasi and DeNicola 2020; Di Benedetto et al. 2021; Kreß et al. 2021).

In OSCC CSCs, mitochondrial dynamics, redox regulation, and metabolic adaptation appear to operate as an integrated network rather than isolated processes. The CD44‐xCT‐NRF2 axis sustains mitochondrial membrane potential and antioxidant defense, whereas high ALDH activity supports detoxification of reactive aldehydes and adaptation to hypoxic niches. These mechanisms converge with DRP1‐mediated mitochondrial fission to maintain bioenergetic flexibility, stemness, and survival under metabolic stress (Ma et al. 2025). Together, these pathways connect mitochondrial quality control with redox homeostasis, thereby contributing to therapeutic resistance and OSCC progression.

Bioenergetic profiling in OSCC may provide clinically relevant insights, as metabolic reprogramming involving glucose, lipid, and amino acid metabolism supports tumor progression and microenvironmental adaptation (Olmedo et al. 2023). Metabolic signatures, including glycolytic and lactate‐related markers, have also been associated with prognosis, metastasis, and therapy response in OSCC (Wang et al. 2022).

Mitochondrial metabolism is also linked to epigenetic regulation, as metabolites such as acetyl‐CoA and α‐ketoglutarate act as cofactors controlling genes related to pluripotency and differentiation (Zhang et al. 2024). Although this relationship remains poorly explored in OSCC CSCs, evidence from other tumors suggests that mitochondrial alterations modulate epigenetic enzymes and affect the expression of stemness‐associated factors, including SOX2, OCT4, and NANOG (Zhang et al. 2024).

Although these mitochondrial mechanisms represent promising therapeutic targets in OSCC CSCs, several pathways discussed in this review are currently supported by a limited number of mechanistic studies and heterogeneous experimental models. In addition, the marked metabolic plasticity of CSCs and the partial extrapolation of evidence from other tumor types indicate that these findings should be interpreted cautiously until validated in larger and translationally relevant studies.

Furthermore, our findings indicate that mitochondria represent promising therapeutic targets in oral cancer. Strategies such as inhibiting mitochondrial fission via DRP1, blocking adaptive mitophagy mediated by BNIP3/PINK1/Parkin, modulating NRF2 signaling, and manipulating lipid metabolism emerge as potential approaches to sensitize CSCs to chemotherapy and radiotherapy (Du et al. 2025).

A major challenge in targeting CSCs through mitochondrial regulation is achieving therapeutic selectivity without affecting normal cells, since OXPHOS and mitochondrial respiration are also essential for normal stem cells and highly energetic tissues, including cardiac and neuronal cells. Moreover, CSC metabolic plasticity may enable adaptive switching between glycolytic and oxidative states under therapeutic pressure, contributing to treatment resistance. Therefore, future strategies should prioritize CSC‐specific metabolic vulnerabilities, such as altered mitochondrial dynamics and redox imbalance, while minimizing off‐target effects.

In summary, targeting mitochondrial dynamics in CSCs offers a promising strategy to overcome therapy resistance and limit OSCC progression, but careful design is essential to exploit CSC‐specific vulnerabilities while preserving normal tissue function.

## 5 Limitations

This review has several important limitations that should be considered when interpreting the findings. First, the current evidence base investigating mitochondrial regulation in CSCs from OSCC remains limited, with only a small number of eligible studies available. Moreover, most studies were conducted predominantly in vitro, frequently using a restricted number of cell lines and experimental conditions, which may not fully reproduce the biological complexity, heterogeneity, and metabolic plasticity of CSCs within native tumor microenvironments. Another important limitation is the heterogeneity among the included studies regarding CSC identification markers, mitochondrial targets, metabolic assays, and experimental methodologies. This variability limits direct comparisons across studies and reduces the overall robustness and reproducibility of the reported findings. In addition, the absence of studies employing immunocompetent animal models, patient‐derived systems, or comprehensive functional analyses of clinical specimens restricts the translational relevance of the available evidence and limits the extrapolation of these findings to human disease. Methodological concerns were also identified, including incomplete reporting of blinding procedures, sample size calculations, and experimental standardization, contributing to a moderate risk of bias across studies. Although the QUIN tool is not specifically designed for molecular and cellular mechanistic studies, it was selected because it provides a structured and reproducible framework for evaluating general methodological quality domains, allowing consistent assessment across the included studies. Importantly, the limited number of studies supporting each mitochondrial mechanism discussed in this review prevents definitive conclusions regarding the universal role of these pathways in OSCC CSC biology. Therefore, the findings presented here should be interpreted as preliminary but biologically relevant evidence that highlights promising mechanistic trends rather than fully established therapeutic paradigms. Future investigations incorporating larger datasets, standardized methodologies, clinically relevant models, and translational validation approaches will be essential to confirm the reproducibility, biological significance, and therapeutic applicability of mitochondrial targeting strategies in OSCC CSCs.

## 6 Conclusion

Evidence shows that OSCC CSCs rely on mitochondrial plasticity to sustain survival, progression, and resistance. Alterations in biogenesis, dynamics, mitophagy, and redox metabolism provide adaptive advantages that hinder eradication. Targets such as CD44‐xCT‐Nrf2, PGC‐1α/ERRα, SIRT1/PGC‐1α, BNIP3, DRP1, and lipid metabolism emerge as strategic intervention points. Therapies that exploit this mitochondrial dependence appear promising, but more preclinical and clinical studies are needed to clarify mitochondria's role in OSCC CSCs.

## Author Contributions

**Rebeca Barbosa da Rocha:** methodology, investigation, validation, visualization, writing – original draft, writing – review and editing. **Jayane da Silva Sousa:** investigation, formal analysis, writing – original draft. **Maria Eduarda de Araujo de Carvalho:** investigation, formal analysis, writing – original draft. **José Delano Barreto Marinho‐Filho:** writing – review and editing, methodology, validation, supervision. **Benedito dos Santos Alves:** methodology, investigation, formal analysis, writing – review and editing. **Rodrigo Elísio de Sá:** conceptualization, methodology, data curation, investigation, validation, formal analysis, writing – original draft, writing – review and editing. **Ana Jérsia Araújo:** conceptualization, methodology, validation, supervision, visualization, project administration, resources, writing – review and editing.

## Funding

The authors have nothing to report.

## Conflicts of Interest

The authors declare no conflicts of interest.

## Supporting information


**Table S1:** Terms of search strategy.
**Table S2:** Search strings.
**Table S3:** Assessment of the quality of in vitro studies using the QUIN tool.
**Table S4:** RoB Bias Risk Assessment (Syrcle's RoB tool).

## Data Availability

The data that support the findings of this study are available from the corresponding author upon reasonable request.

## References

[odi70449-bib-0001] Ahmad, P. , R. Nawaz , M. Qurban , et al. 2021. “Risk Factors Associated With the Mortality Rate of Oral Squamous Cell Carcinoma Patients: A 10‐Year Retrospective Study.” Medicine 100, no. 36: e27127. 10.1097/MD.0000000000027127. PMC8428756 34516504

[odi70449-bib-0002] Askari, H. , F. Rabiei , F. Lohrasbi , S. Ghadir , A. Mehdipour Arbastan , and M. Ghasemi‐Kasman . 2024. “AMP‐Activated Protein Kinase as a Mediator of Mitochondrial Dysfunction of Multiple Sclerosis in Animal Models: A Systematic Review.” Journal of Cellular Physiology 239, no. 5: e31230. 10.1002/jcp.31230. 38403972

[odi70449-bib-0003] Bossi, P. , S. Lo Vullo , M. Guzzo , et al. 2014. “Preoperative Chemotherapy in Advanced Resectable OCSCC: Long‐Term Results of a Randomized Phase III Trial.” Annals of Oncology: Official Journal of the European Society for Medical Oncology 25, no. 2: 462–466. 10.1093/annonc/mdt555. 24401930

[odi70449-bib-0004] Chamoli, A. , A. S. Gosavi , U. P. Shirwadkar , et al. 2021. “Overview of Oral Cavity Squamous Cell Carcinoma: Risk Factors, Mechanisms, and Diagnostics.” Oral Oncology 121: 105451. 10.1016/j.oraloncology.2021.105451. 34329869

[odi70449-bib-0005] Chen, W. , H. Zhao , and Y. Li . 2023. “Mitochondrial Dynamics in Health and Disease: Mechanisms and Potential Targets.” Signal Transduction and Targeted Therapy 8, no. 1: 333. 10.1038/s41392-023-01547-9. PMC10480456 37669960

[odi70449-bib-0006] DeBlasi, J. M. , and G. M. DeNicola . 2020. “Dissecting the Crosstalk Between NRF2 Signaling and Metabolic Processes in Cancer.” Cancers 12, no. 10: 3023. 10.3390/cancers12103023. PMC7603127 33080927

[odi70449-bib-0007] Dhumal, S. N. , S. K. Choudhari , S. Patankar , S. S. Ghule , Y. B. Jadhav , and S. Masne . 2022. “Cancer Stem Cell Markers, CD44 and ALDH1, for Assessment of Cancer Risk in OPMDs and Lymph Node Metastasis in Oral Squamous Cell Carcinoma.” Head and Neck Pathology 16: 453–465. 10.1007/s12105-021-01384-8. PMC9187836 34655409

[odi70449-bib-0008] Di Benedetto, G. , S. Parisi , T. Russo , and F. Passaro . 2021. “YAP and TAZ Mediators at the Crossroad Between Metabolic and Cellular Reprogramming.” Metabolites 11, no. 3: 154. 10.3390/metabo11030154. PMC7999074 33800464

[odi70449-bib-0009] Di Gregorio, J. , S. Petricca , R. Iorio , E. Toniato , and V. Flati . 2022. “Mitochondrial and Metabolic Alterations in Cancer Cells.” European Journal of Cell Biology 101, no. 3: 151225. 10.1016/j.ejcb.2022.151225. 35453093

[odi70449-bib-0010] Du, H. , T. Xu , S. Yu , S. Wu , and J. Zhang . 2025. “Mitochondrial Metabolism and Cancer Therapeutic Innovation.” Signal Transduction and Targeted Therapy 10, no. 1: 245. 10.1038/s41392-025-02311-x. PMC12319113 40754534

[odi70449-bib-0011] Garimella, S. V. , S. C. Gampa , and P. Chaturvedi . 2023. “Mitochondria in Cancer Stem Cells: From an Innocent Bystander to a Central Player in Therapy Resistance.” Stem Cells and Cloning: Advances and Applications 16: 19–41. 10.2147/SCCAA.S417842. PMC10460581 37641714

[odi70449-bib-0012] Heawchaiyaphum, C. , H. Yoshiyama , H. Iizasa , et al. 2023. “Epstein‐Barr Virus Promotes Oral Squamous Cell Carcinoma Stemness Through the Warburg Effect.” International Journal of Molecular Sciences 24, no. 18: 14072. 10.3390/ijms241814072. PMC10531857 37762374

[odi70449-bib-0013] Hooijmans, C. R. , M. M. Rovers , R. B. M. de Vries , M. Leenaars , M. Ritskes‐Hoitinga , and M. W. Langendam . 2014. “SYRCLE's Risk of Bias Tool for Animal Studies.” BMC Medical Research Methodology 14: 43. 10.1186/1471-2288-14-43. PMC4230647 24667063

[odi70449-bib-0014] Huang, A. , H. Xue , T. Xie , et al. 2025. “A Review of the Pathogenesis of Mitochondria in Breast Cancer and Progress of Targeting Mitochondria for Breast Cancer Treatment.” Journal of Translational Medicine 23, no. 1: 70. 10.1186/s12967-025-06077-2. PMC11734335 39815317

[odi70449-bib-0015] Jagust, P. , B. de Luxán‐Delgado , B. Parejo‐Alonso , and P. Sancho . 2019. “Metabolism‐Based Therapeutic Strategies Targeting Cancer Stem Cells.” Frontiers in Pharmacology 10: 203. 10.3389/fphar.2019.00203. PMC6438930 30967773

[odi70449-bib-0016] Jin, P. , J. Jiang , L. Zhou , et al. 2022. “Mitochondrial Adaptation in Cancer Drug Resistance: Prevalence, Mechanisms, and Management.” Journal of Hematology & Oncology 15, no. 1: 97. 10.1186/s13045-022-01313-4. PMC9290242 35851420

[odi70449-bib-0017] Kowalczyk, P. , D. Sulejczak , P. Kleczkowska , et al. 2021. “Mitochondrial Oxidative Stress‐A Causative Factor and Therapeutic Target in Many Diseases.” International Journal of Molecular Sciences 22, no. 24: 13384. 10.3390/ijms222413384. PMC8707347 34948180

[odi70449-bib-0018] Kreß, J. K. C. , C. Jessen , A. Marquardt , A. Hufnagel , and S. Meierjohann . 2021. “NRF2 Enables EGFR Signaling in Melanoma Cells.” International Journal of Molecular Sciences 22, no. 8: 3803. 10.3390/ijms22083803. PMC8067606 33916908

[odi70449-bib-0019] Li, X. , H. S. Chaouhan , S.‐H. Yu , et al. 2025. “Hypoxia‐Induced Metabolic and Functional Changes in Oral CSCs: Implications for Stemness and Viability Modulation Through BNIP3‐Driven Mitophagy.” Journal of Cellular and Molecular Medicine 29, no. 3: e70400. 10.1111/jcmm.70400. PMC11822456 39945227

[odi70449-bib-0020] Lin, Q. , J. Chen , L. Gu , X. Dan , C. Zhang , and Y. Yang . 2021. “New Insights Into Mitophagy and Stem Cells.” Stem Cell Research & Therapy 12, no. 1: 452. 10.1186/s13287-021-02520-5. PMC8359610 34380561

[odi70449-bib-0021] Ma, N. , Y. Wang , X. Li , M. Xu , and D. Tan . 2025. “Reactive Oxygen Species in Cancer: Mechanistic Insights and Therapeutic Innovations.” Cell Stress and Chaperones 30: 100108. 10.1016/j.cstres.2025.100108. PMC12398932 40769273

[odi70449-bib-0022] Miao, Y. , P. Wang , J. Huang , et al. 2024. “Metabolomics, Transcriptome and Single‐Cell RNA Sequencing Analysis of the Metabolic Heterogeneity Between Oral Cancer Stem Cells and Differentiated Cancer Cells.” Cancers 16, no. 2: 237. 10.3390/cancers16020237. PMC10813553 38254728

[odi70449-bib-0023] Mohamad, I. , M. D. E. Glaun , K. Prabhash , et al. 2023. “Current Treatment Strategies and Risk Stratification for Oral Carcinoma.” American Society of Clinical Oncology Educational Book. American Society of Clinical Oncology. Annual Meeting 43: e389810. 10.1200/EDBK_389810. 37200591

[odi70449-bib-0024] Napolitano, G. , G. Fasciolo , and P. Venditti . 2021. “Mitochondrial Management of Reactive Oxygen Species.” Antioxidants 10, no. 11: 1824. 10.3390/antiox10111824. PMC8614740 34829696

[odi70449-bib-0025] Olmedo, I. , D. Martínez , J. Carrasco‐Rojas , and J. A. Jara . 2023. “Mitochondria in Oral Cancer Stem Cells: Unraveling the Potential Drug Targets for New and Old Drugs.” Life Sciences 331: 122065. 10.1016/j.lfs.2023.122065. 37659591

[odi70449-bib-0026] Onishi, M. , K. Yamano , M. Sato , N. Matsuda , and K. Okamoto . 2021. “Molecular Mechanisms and Physiological Functions of Mitophagy.” EMBO Journal 40, no. 3: e104705. 10.15252/embj.2020104705. PMC7849173 33438778

[odi70449-bib-0027] Page, M. J. , D. Moher , P. M. Bossuyt , et al. 2021. “PRISMA 2020 Explanation and Elaboration: Updated Guidance and Exemplars for Reporting Systematic Reviews.” BMJ (Clinical Research Ed.) 372: n160. 10.1136/bmj.n160. PMC8005925 33781993

[odi70449-bib-0028] Phi, L. T. H. , I. N. Sari , Y.‐G. Yang , et al. 2018. “Cancer Stem Cells (CSCs) in Drug Resistance and Their Therapeutic Implications in Cancer Treatment.” Stem Cells International 2018: 5416923. 10.1155/2018/5416923. PMC5850899 29681949

[odi70449-bib-0029] Praharaj, P. P. , A. Singh , S. Patra , and S. K. Bhutia . 2023. “Co‐Targeting Autophagy and NRF2 Signaling Triggers Mitochondrial Superoxide to Sensitize Oral Cancer Stem Cells for Cisplatin‐Induced Apoptosis.” Free Radical Biology & Medicine 207: 72–88. 10.1016/j.freeradbiomed.2023.07.008. 37423560

[odi70449-bib-0030] Sá, R. E. , B. O. Almeida , M. C. Mota , et al. 2026. “Dual Inhibition of MMP‐2 and Actin Dynamics by a Novel Bis‐Chalcone: An Anticancer Strategy for Oral Squamous Cell Carcinoma.” Naunyn‐Schmiedeberg's Archives of Pharmacology 399: 11643. 10.1007/s00210-026-05050-0. PMC13269452 41721121

[odi70449-bib-0031] Saluja, T. S. , V. Kumar , M. Agrawal , et al. 2020. “Mitochondrial Stress–Mediated Targeting of Quiescent Cancer Stem Cells in Oral Squamous Cell Carcinoma.” Cancer Management and Research 12: 4519–4530. 10.2147/CMAR.S252292. PMC7305346 32606945

[odi70449-bib-0032] Sheng, X. , M.‐M. Wang , G.‐D. Zhang , et al. 2024. “Dual Inhibition of Oxidative Phosphorylation and Glycolysis to Enhance Cancer Therapy.” Bioorganic Chemistry 147: 107325. 10.1016/j.bioorg.2024.107325. 38583247

[odi70449-bib-0033] Sheth, V. H. , N. P. Shah , R. Jain , N. Bhanushali , and V. Bhatnagar . 2024. “Development and Validation of a Risk‐Of‐Bias Tool for Assessing *in Vitro* Studies Conducted in Dentistry: The QUIN.” Journal of Prosthetic Dentistry 131, no. 6: 1038–1042. 10.1016/j.prosdent.2022.05.019. 35752496

[odi70449-bib-0034] Shibata, M. , and M. O. Hoque . 2019. “Targeting Cancer Stem Cells: A Strategy for Effective Eradication of Cancer.” Cancers 11, no. 5: 732. 10.3390/cancers11050732. PMC6562442 31137841

[odi70449-bib-0035] Shimonosono, M. , K. Tanaka , S. Flashner , et al. 2021. “Alcohol Metabolism Enriches Squamous Cell Carcinoma Cancer Stem Cells That Survive Oxidative Stress via Autophagy.” Biomolecules 11, no. 10: 1479. 10.3390/biom11101479. PMC8533166 34680112

[odi70449-bib-0036] Shin, Y. Y. , Y. Seo , S.‐J. Oh , et al. 2022. “Melatonin and Verteporfin Synergistically Suppress the Growth and Stemness of Head and Neck Squamous Cell Carcinoma Through the Regulation of Mitochondrial Dynamics.” Journal of Pineal Research 72, no. 1: e12779. 10.1111/jpi.12779. 34826168

[odi70449-bib-0037] Solomon, B. , R. J. Young , and D. Rischin . 2018. “Head and Neck Squamous Cell Carcinoma: Genomics and Emerging Biomarkers for Immunomodulatory Cancer Treatments.” Seminars in Cancer Biology 52, no. Pt 2: 228–240. 10.1016/j.semcancer.2018.01.008. 29355614

[odi70449-bib-0038] Tan, E. W. , A. D. I. Abdullah , L. C. Ming , et al. 2024. “Adenovirus‐Mediated Expression of MOAP‐1, Bax and RASSF1A Antagonizes Chemo‐Drug Resistance of Human Breast Cancer Cells Expressing Cancer Stem Cell Markers.” Biomedicine & Pharmacotherapy 176: 116744. 10.1016/j.biopha.2024.116744. 38810399

[odi70449-bib-0039] Tan, E. W. , S. K. Singh , K. Dua , et al. 2025. “Cancer Stem Cells: Mitochondria Signalling Pathway and Strategies for Therapeutic Interventions.” Molecular Biology Reports 52, no. 1: 671. 10.1007/s11033-025-10748-0. PMC12226654 40608151

[odi70449-bib-0040] Vasan, K. , M. Werner , and N. S. Chandel . 2020. “Mitochondrial Metabolism as a Target for Cancer Therapy.” Cell Metabolism 32, no. 3: 341–352. 10.1016/j.cmet.2020.06.019. PMC7483781 32668195

[odi70449-bib-0041] Wang, Y. , X. Zhang , S. Wang , et al. 2022. “Identification of Metabolism‐Associated Biomarkers for Early and Precise Diagnosis of Oral Squamous Cell Carcinoma.” Biomolecules 12, no. 3: 400. 10.3390/biom12030400. PMC8945702 35327590

[odi70449-bib-0042] Wang, Z. , S. Tang , L. Cai , et al. 2024. “DRP1 Inhibition‐Mediated Mitochondrial Elongation Abolishes Cancer Stemness, Enhances Glutaminolysis, and Drives Ferroptosis in Oral Squamous Cell Carcinoma.” British Journal of Cancer 130, no. 11: 1744–1757. 10.1038/s41416-024-02670-2. PMC11130175 38582810

[odi70449-bib-0043] Weckx, A. , K. J. Grochau , A. Grandoch , T. Backhaus , J. E. Zöller , and M. Kreppel . 2020. “Survival Outcomes After Surgical Treatment of Oral Squamous Cell Carcinoma.” Oral Diseases 26, no. 7: 1432–1439. 10.1111/odi.13422. 32428375

[odi70449-bib-0044] Wu, F. , S. Chen , S. Ren , et al. 2024. “Regulating Lipid Metabolism via Mitochondrial Dynamics in Tongue Squamous Cell Carcinoma Cancer Stem Cells.” Recent Patents on Anti‐Cancer Drug Discovery 20: 445–459. 10.2174/0115748928275772231226063458. 38305307

[odi70449-bib-0045] Wu, Z. , C. Xiao , F. Li , W. Huang , F. You , and X. Li . 2024. “Mitochondrial Fusion‐Fission Dynamics and Its Involvement in Colorectal Cancer.” Molecular Oncology 18, no. 5: 1058–1075. 10.1002/1878-0261.13578. PMC11076987 38158734

[odi70449-bib-0046] Yuan, S.‐S. F. , Y.‐M. Wang , L.‐P. Chan , et al. 2023. “IL‐1RA Promotes Oral Squamous Cell Carcinoma Malignancy Through Mitochondrial Metabolism‐Mediated EGFR/JNK/SOX2 Pathway.” Journal of Translational Medicine 21, no. 1: 473. 10.1186/s12967-023-04343-9. PMC10351194 37461111

[odi70449-bib-0047] Zhang, C. , Y. Meng , and J. Han . 2024. “Emerging Roles of Mitochondrial Functions and Epigenetic Changes in the Modulation of Stem Cell Fate.” Cellular and Molecular Life Sciences: CMLS 81, no. 1: 26. 10.1007/s00018-023-05070-6. PMC11072137 38212548

[odi70449-bib-0048] Zhang, Y. , B. Yang , C. Tu , et al. 2023. “Mitochondrial Impairment and Downregulation of Drp1 Phosphorylation Underlie the Antiproliferative and Proapoptotic Effects of Alantolactone on Oral Squamous Cell Carcinoma Cells.” Journal of Translational Medicine 21, no. 1: 328. 10.1186/s12967-023-04188-2. PMC10193726 37198593

