# Supplementary material for: Mapping the Bioenergetic Landscape of Oral Cancer Stem Cells: A Systematic Review
Source: Oral Dis. 2026 Aug 3;32(10):3089–100. doi: 10.1111/odi.70449 (PMC13768945; doi:10.1111/odi.70449)
Supplement: Supplementary file 1 [file ODI-32-3089-s001.docx]

**Supplementary Material**

**MAPPING THE BIOENERGETIC LANDSCAPE OF ORAL CANCER STEM CELLS: A SYSTEMATIC REVIEW**

*Mitochondrial regulation of cancer stem cells*

*Rodrigo Elísio de Sá^a^, Rebeca Barbosa da Rocha^a^, Benedito Dos Santos Alves^a^, Maria Eduarda de Araujo de Carvalho^a^, Jayane da Silva Sousa^a^, José Delano Barreto Marinho Filho^a^, Ana Jérsia Araújo^a*^*

*^a^Laboratório de Cultura de Células do Delta (LCCDelta), Universidade Federal Delta do Parnaíba, UFDPar, Parnaíba, PI, Brazil*

** Corresponding author*

Ana Jérsia Araújo, PhD. Laboratório de Cultura de Células do Delta (LCCDelta), Universidade Federal do Delta de Parnaíba, UFDPar, 64202-020, Parnaíba, Brazil. Tel: +55 86 99439 9223. E-mail address: [anajersia@ufdpar.edu.br](mailto:anajersia@ufdpar.edu.br).

**Table S1.** Terms of search strategy.

| **Keywords** | **Controlled Vocabulary** | **MeSH Unique ID** |
| --- | --- | --- |
| Oral squamous cell carcinoma | Yes | D000077195 |
| Oral cancer | No | - |
| OSCC | No | - |
| Cancer stem cell | Yes | D014411 |
| Tumor stem cell | Yes | D014411 |
| CSC | No | - |
| Mitochondria | Yes | D008928 |
| Mitochondrial dynamics | Yes | D063154 |
| Drug target | Yes | D016503 |
| Drug repositioning | Yes | D058492 |
| Repurposed drug | Yes | D058492 |
| Therapeutic target | No | - |
| Oxidative phosphorylation | Yes | D010085 |
| OXPHOS | No | - |

**Table S2.** Search strings.

| **Databases** | **Search strings** |
| --- | --- |
| **PubMed** | ((((((("oral squamous cell carcinoma"[Title/Abstract]) OR ("oral cancer"[Title/Abstract])) OR ("OSCC"[Title/Abstract])) OR ("cancer stem cell*"[Title/Abstract])) OR ("tumor stem cell*"[Title/Abstract])) OR ("CSC"[Title/Abstract])) AND (((mitochondria[Title/Abstract]) OR ("mitochondrial metabolism"[Title/Abstract])) OR ("mitochondrial dynamics"[Title/Abstract]))) AND (((((("drug target*"[Title/Abstract]) OR ("drug repositioning"[Title/Abstract])) OR ("repurposed drug*"[Title/Abstract])) OR ("therapeutic target*"[Title/Abstract])) OR ("oxidative phosphorylation"[Title/Abstract])) OR (OXPHOS[Title/Abstract])) |
| **Embase** | #1 'oral squamous cell carcinoma':ab,ti OR 'oral cancer':ab,ti OR oscc:ab,ti OR 'cancer stem cell*':ab,ti OR 'tumor stem cell*':ab,ti OR csc:ab,ti  AND  #2 'mitochondria':ab,ti OR 'mitochondrial metabolism':ab,ti OR 'mitochondrial dynamics':ab,ti  AND  #3 'drug target*':ab,ti OR 'drug repositioning':ab,ti OR 'repurposed drug*':ab,ti OR 'therapeutic target*':ab,ti OR 'oxidative phosphorylation':ab,ti OR 'oxphos':ab,ti |
| **Scopus** | ( ( TITLE-ABS-KEY ( "oral squamous cell carcinoma" ) OR TITLE-ABS-KEY ( "oral cancer" ) OR TITLE-ABS-KEY ( "cancer stem cell*" ) OR TITLE-ABS-KEY ( "tumor stem cell*" ) OR TITLE-ABS-KEY ( CSC ) ) ) AND ( ( TITLE-ABS-KEY ( mitochondria ) OR TITLE-ABS-KEY ( "mitochondrial metabolism" ) OR TITLE-ABS-KEY ( "mitochondrial dynamics" ) ) ) AND ( ( TITLE-ABS-KEY ( "drug target*" ) OR TITLE-ABS-KEY ( "drug repositioning" ) OR TITLE-ABS-KEY ( "repurposed drug*" ) OR TITLE-ABS-KEY ( "therapeutic target*" ) OR TITLE-ABS-KEY ( "oxidative phosphorylation" ) OR TITLE-ABS-KEY ( OXPHOS ) ) ) |
| **Web of science** | #1 "oral squamous cell carcinoma" (Title) or "oral cancer" (Title) or OSCC (Title) or "cancer stem cell*" (Title) or "tumor stem cell*" (Title) or CSC (Title)  AND  #2 "mitochondria" (Title) or "mitochondrial metabolism" (Title) or "mitochondrial dynamics" (Title)  AND  #3"drug target*" (Title) or "drug repositioning" (Title) or "repurposed drug*" (Title) or "therapeutic target*" (Title) or "oxidative phosphorylation" (Title) or OXPHOS (Title) |

**Table S3** Assessment of the quality of *in vitro* studies using the QUIN tool.

| **Study/ Year** | **QUIN Item** | | | | | | | | | | | | | |
| --- | --- | --- | --- | --- | --- | --- | --- | --- | --- | --- | --- | --- | --- | --- |
|  | **D1** | **D2** | **D3** | **D4** | **D5** | **D6** | **D7** | **D8** | **D9** | **D10** | **D11** | **D12** | **Risk of Bias*** |  |
| Wu et al. 2023 | 2 | 0 | 1 | 2 | 2 | 0 | 0 | 2 | 1 | 0 | 2 | 2 | Medium |  |
| Li et al. 2025 | 2 | 0 | 1 | 2 | 2 | 0 | 0 | 2 | 1 | 0 | 2 | 2 | Medium |  |
| Shimonosono et al. 2021 | 2 | 0 | 1 | 2 | 2 | 0 | 0 | 2 | 1 | 0 | 2 | 2 | Medium |  |
| Wang et al. 2024 | 2 | 0 | 1 | 2 | 2 | 0 | 0 | 2 | 1 | 0 | 2 | 2 | Medium |  |
| Shin et al. 2021 | 2 | 0 | 1 | 2 | 2 | 0 | 0 | 2 | 1 | 0 | 2 | 2 | Medium |  |
| Saluja et al. 2020 | 2 | 0 | 1 | 2 | 2 | 0 | 0 | 2 | 1 | 0 | 2 | 2 | Medium |  |
| Praharaj et al. 2023  Yuan et al. 2023 | 2  2 | 0  0 | 1  1 | 2  2 | 2  2 | 0  0 | 0  0 | 2  2 | 1  1 | 0  0 | 2  2 | 2  2 | Medium  Medium |  |
| Miao et al. 2024 | 2 | 1 | 2 | 2 | 2 | 1 | 1 | 2 | 1 | 0 | 2 | 2 | Low |  |

*Score: adequately specified=2; inadequately specified=1; not specified (NS)=0; not applicable (NA). Final Score: total score 100/2 number of criteria applicable.>70%=low risk of bias;50–70%=medium risk of bias; and <50%=high risk of bias

**Table S4.** RoB Bias Risk Assessment (Syrcle’s RoB tool).

| **Study/Year** | **Syrcle Item** | | | | | | | | | | |
| --- | --- | --- | --- | --- | --- | --- | --- | --- | --- | --- | --- |
|  | **D1** | **D2** | **D3** | **D4** | **D5** | **D6** | **D7** | **D8** | **D9** | **D10** |  |
| Yuan et al. 2023 [31] | No | No | UR | UR | Yes | UR | Yes | No | No | UR |  |
| Heawchaiyaphum et al. 2023 [24] | UR | No | UR | UR | Yes | UR | Yes | No | No | UR |  |
| Shin et al. 2021 [29] | UR | No | UR | UR | UR | UR | UR | No | No | UR |  |
| Shimonosono et al. 2021 [27] | UR | No | UR | UR | Yes | UR | Yes | No | No | UR |  |

*Score: No: low risk of bias; UR: uncertain risk; Yes: high risk of bias.
